# In vitro characterization of odorranalectin for peptide-based drug delivery across the blood–brain barrier

**DOI:** 10.1186/s12868-019-0504-x

**Published:** 2019-05-08

**Authors:** Ravi K. Sajja, Predrag Cudic, Luca Cucullo

**Affiliations:** 1 Department of Pharmaceutical Sciences, Texas Tech University Health Sciences Center, 1300 S. Coulter Street, Amarillo, TX 79106 USA; 2 Department of Chemistry and Biochemistry, Florida Atlantic University, Boca Raton, FL USA

**Keywords:** siRNA delivery, BBB, CNS disorders, Cyclic peptide, hCMEC/D3

## Abstract

**Background:**

The use of siRNA-based gene silencing has been recently underscored as a potential therapeutic strategy for the treatment of neurological disorders. However, the stability of siRNA and other small molecule therapeutics is challenged by their intrinsic instability and limited passage across the blood–brain barrier (BBB). Based on these premises, our objective was to characterize/optimize odorranalectin (OL), a small non-immunogenic lectin-like peptide, as a carrier for targeted delivery across the BBB. For this purpose, 5(6)-carboxyfluorescein-conjugated OL and scramble peptide were synthesized, and then their BBB cellular internalization/trafficking and stability were characterized versus temperature, pH and serum content in the media in hCMEC/D3 cells as a model of BBB endothelium. Specifically, integrity of the internalized peptide in cell lysates was analyzed by LC/MS while cellular distribution and intracellular trafficking of OL was examined by fluorescence microscopy with early-late endosome (pHRodo Red^®^) and lysosome (Lysotracker^®^) markers.

**Results:**

Our data show that cellular uptake of OL increased linearly with the concentrations tested in this study at 37 °C and the uptake was two to threefolds higher when compared to scramble peptide. While there were no differences for scramble peptide, the uptake of OL decreased by 50% at 4 °C incubation (vs. 37 °C). No effects of pH were observed on endothelial uptake of OL. Immunofluorescence studies also indicated a significant cellular internalization of OL that remained intact (as evaluated by LC–MS/MS) and co-localized with endosomal, but not lysosome marker. Importantly, OL was found non-toxic to cells at all concentrations tested.

**Conclusions:**

In summary, our data suggest the existence of a receptor-mediated transcytosis pathway for cellular uptake of OL at the BBB endothelium. However, in vivo studies will be needed to assess the siRNA loading capacity of OL and its trans-BBB transport efficiency for targeted delivery in the brain.

## Background

While the BBB is critical to maintain the homeostasis of the central nervous system (CNS), it is also a major bottleneck for drug delivery to brain [1–3]. Recently, Srimanee et al. showed the potential of cell penetrating peptides for targeted siRNA delivery to glioblastomas across the BBB. Results showed improved gene silencing efficiency associated with this targeted delivery method [3].

Previous studies reported the potential of lectins as drug carriers to specific sites of action based on their ability to selectively bind to specific sugar moieties of targeted cells [4, 5]. In addition, lectins could promote drug targeting into or across the cell barriers through vesicular transport [4]. Our previous studies using BSA-conjugated monosaccharides and cell-based assays showed that FAM-OL strongly binds to L-fucose and to a lesser degree, to galactose and *N*-acetyl-d-galactosamine [6]. However, no binding was observed with d-glucose, *N*-acetyl-d-glucosamine, and *N*-acetyl-d-neuranimic acid. Furthermore, their inherent immunogenicity and toxicity risks limit their use as drug delivery vehicles [4, 7].

OL, isolated from frog skin secretions, is a small lectin-mimicking cyclic peptide (composed of 17 amino acids) with low immunogenicity and was shown to have low toxicity profile in mice [7]. Various studies demonstrated the targeting efficiency of OL for therapeutic delivery to brain for the treatment of CNS diseases [8–10] via binding to sugar complexes at cell membranes [6]. However, no mechanistic proof-of-principle study exists to demonstrate the uptake and the fate of OL into BBB endothelial cells. Therefore, in this study, we demonstrated the cellular uptake mechanisms of OL in hCMEC/D3 cell line, an established in vitro model of human BBB [11–13].

## Results

### Cellular uptake of FAM-OL peptide in BBB endothelial cells

**Fig. 1:**
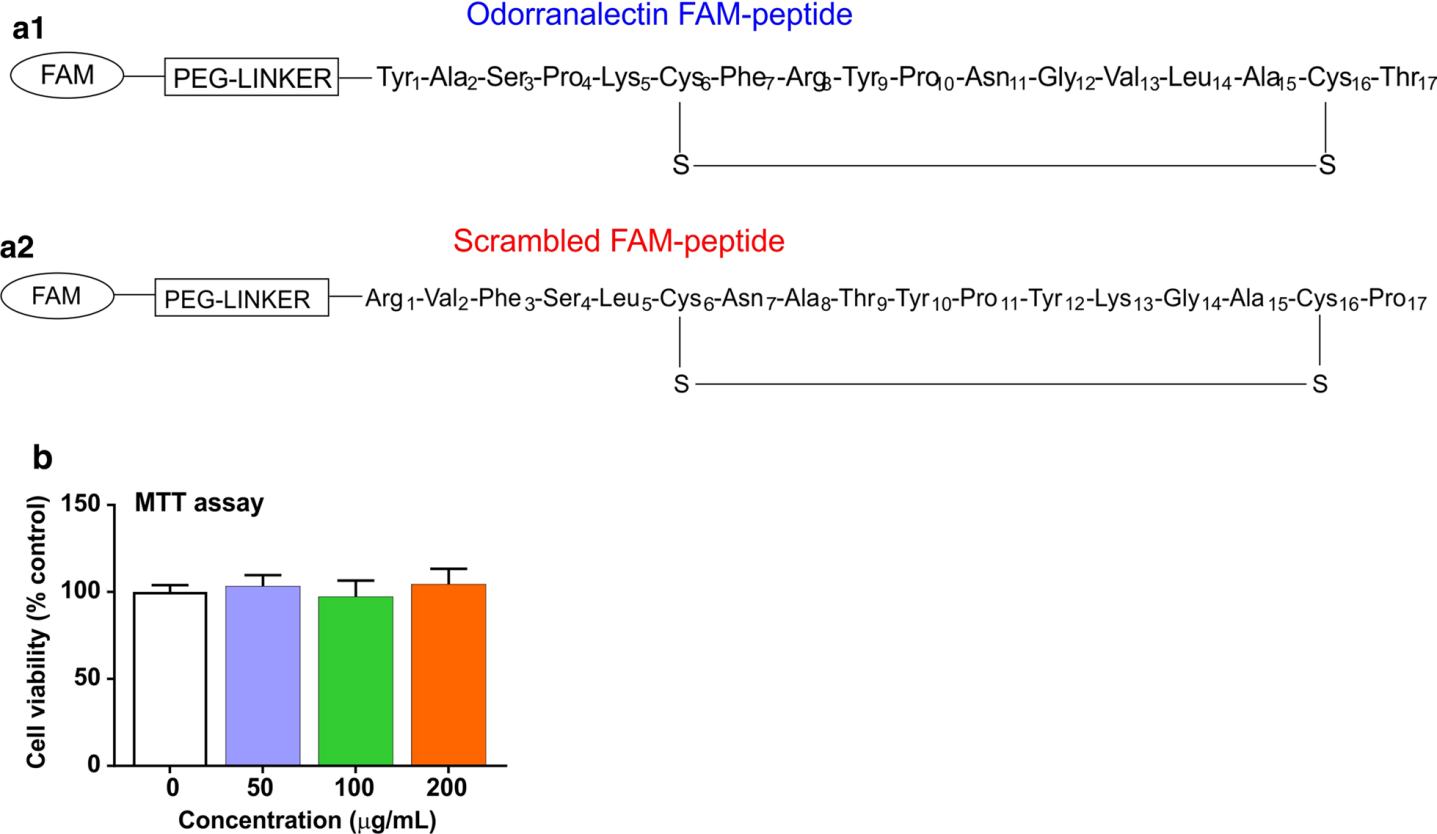
Structure of Odorranalectin peptide and cell viability: **a1** Chemical structure of the OL peptide conjugated with FAM. **a2** Chemical structure of scrambled OL peptide conjugated with FAM moiety. **b** Effects of FAM-OL peptide on hCMEC/D3 cell viability (%) as determined by MTT assay (N = 4–5 biological replicates/group pooled from two independent experiments were used for statistical analyses)

**Fig. 2:**
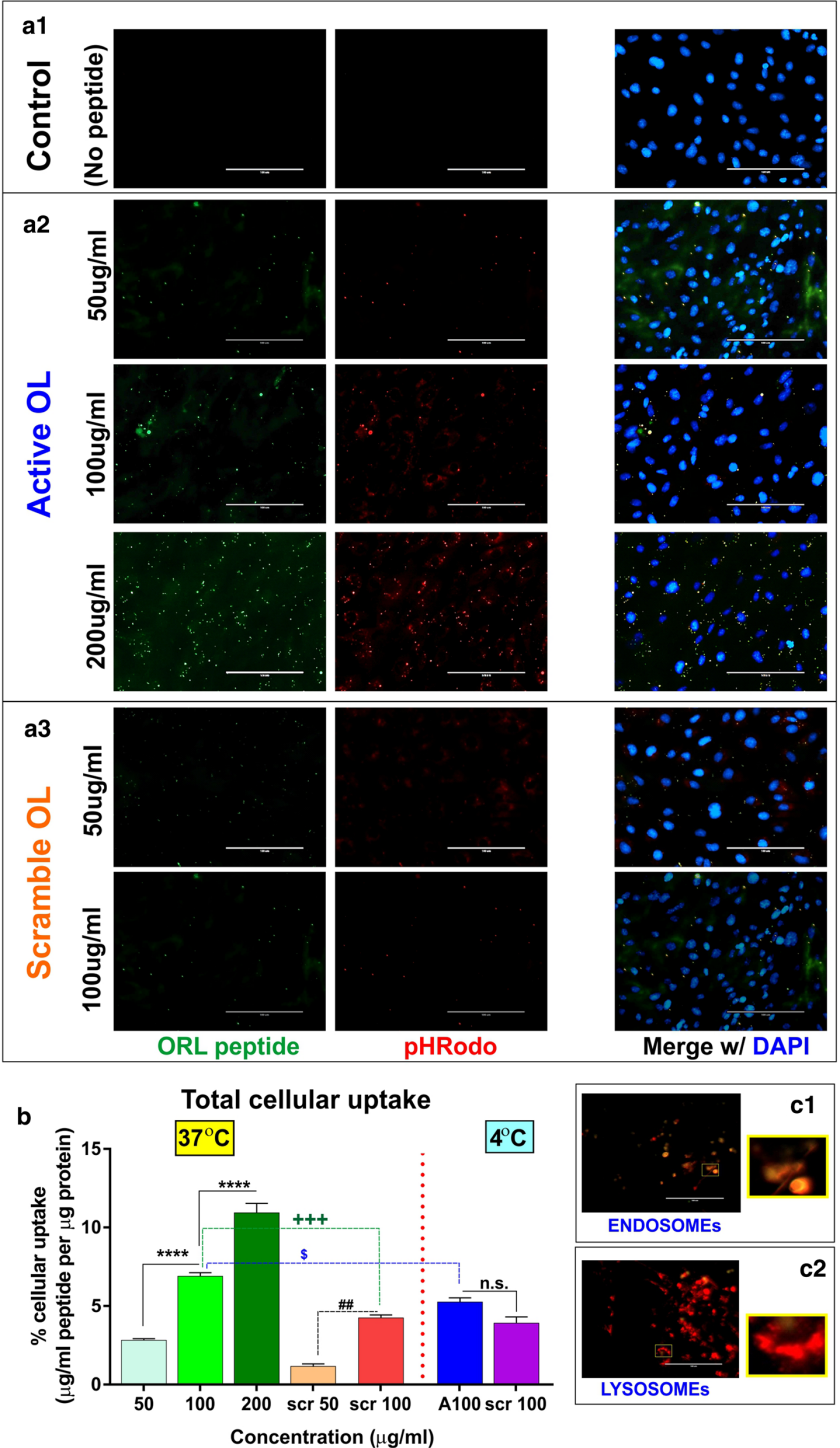
Internalization of OL peptide in hCMEC/D3 cells. **a1**–**a3** Concentration-dependent increase in the cellular uptake of FAM-OL peptide (green) and endosomal marker, pHRodo Red merged with DAPI (blue) versus scrambled peptide. **b** Cellular uptake of FAM-OL peptide and scrambled peptide at 37 °C and 4 °C as determined from the total cell lysates and normalized per µg protein. **c1** Co-localization of the FAM-OL peptide with endosomal (pHRhodo Red) and **c2** lysosomal markers (LysoTracker^®^ Red). Images captured at 40 × (scale = 100 µm) were further magnified in the insets. ****P < 0.0001 versus different concentrations of active peptide; ^##^*P* < 0.01 scramble peptide versus scrambled peptide at different concentrations (50 vs. 100 µg/mL); ^**+++**^*P* < 0.001 active versus scrambled peptide at 100 µg/mL concentrations; ^$^*P* < 0.05 versus 4 °C at 100 µg/mL concentrations. All images were originally captured at 40X (N = 4–5 biological replicates/group pooled from two independent experiments were used for statistical analyses)

As shown in Fig. 1b, FAM-OL peptide tested in the concentration range (0–200 µg/mL) did not affect the viability of hCMEC/D3 endothelial cells. Immunofluorescence analysis showed a concentration-dependent increase in cellular uptake of FAM-OL peptide in hCMEC/D3 cells following 3 h incubation at 37 °C (Fig. 2a1–a3). Our results further demonstrated a co-localization of the peptide with endosomal marker, thus indicating the mode of internalization of the peptide. We next assessed the internalization specificity of the OL peptide by using a scrambled OL peptide sequence as a control. As illustrated in Fig. 2b, significant differences were observed in the uptake of the OL active peptide at 37 °C when compared to identical concentrations of the scrambled peptide (side by side measurements; *P* < 0.01). Interestingly, when measured at 4 °C, the cellular uptake of scrambled OL peptide at 100 µg/mL concentration was not statistically different than that of the active one although it is important to note that the internalization of the active peptide at 4 °C was significantly lower than that measured at 37 °C (*P* < 0.05).

Additionally, our results demonstrated the course and fate of internalized OL-peptide by using organelle trackers. As shown in Fig. 2c1, cellular imaging demonstrated a co-localization of the internalized FAM-OL peptide (green) with the pHRodo red (a marker for early to late endosomes), while there was no co-localization of the peptide with LysoTracker^®^ Red, a lysosomal tracker (Fig. 2c2). This further suggests that peptide may not undergo the lysosomal trapping and degradation.

### Effects of pH and serum concentration on BBB endothelial uptake of OL peptide

**Fig. 3:**
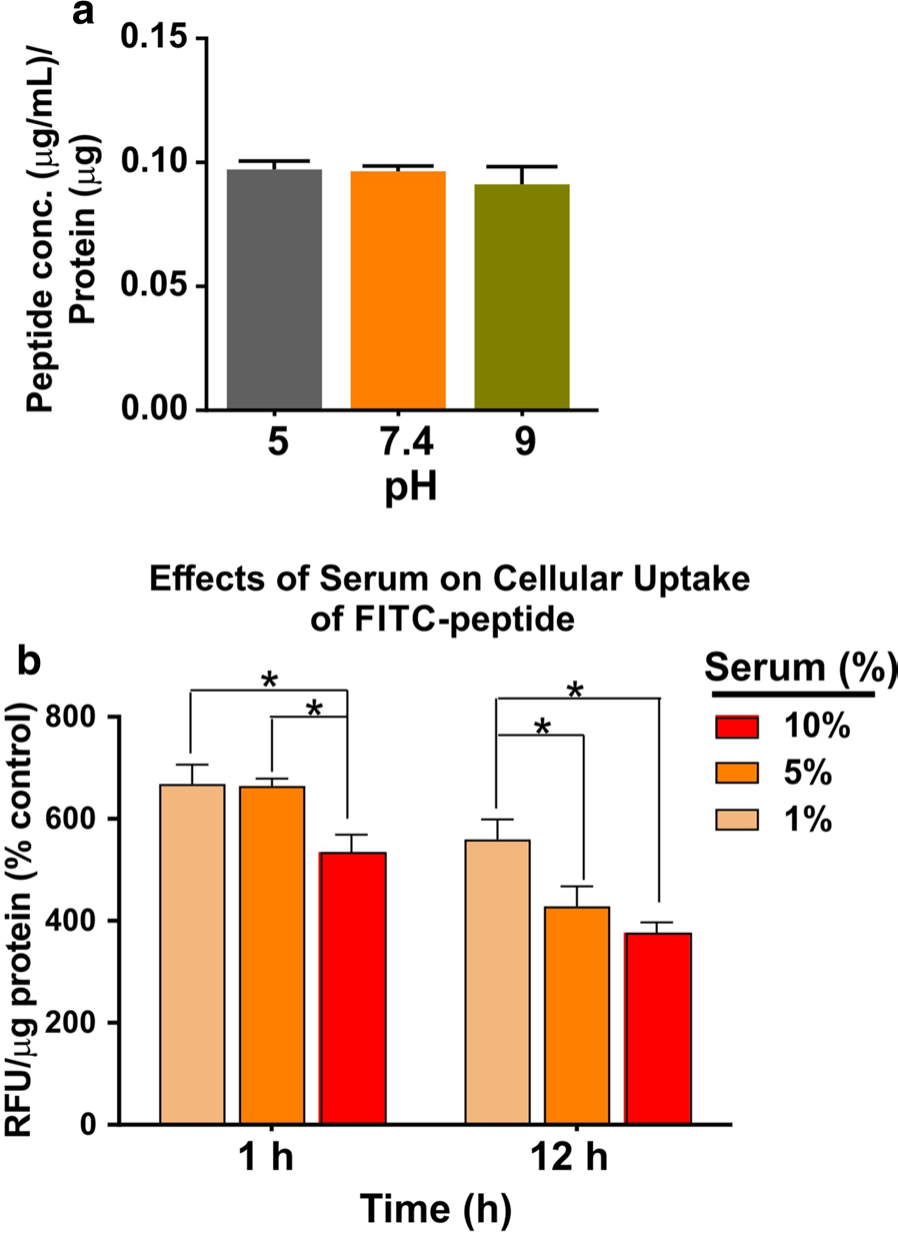
Effects of pH (**a**) and serum % (**b**) of the culture media on the cellular uptake of OL peptide. *RFU* relative florescence units; *p < 0.05. FAM-OL peptide was added at 10 µg/mL and 0.1% DMSO was used as baseline control (N = 3 biological replicates/group pooled from two independent experiments were used for statistical analyses)

As demonstrated in Fig. 3, we determined the impact of pH and serum content (in %) in culture medium on the internalization of the peptide in hCMEC/D3 cells. Change in pH of the medium did not affect the cellular uptake of OL peptide (Fig. 3a). However, as analyzed by one-way ANOVA followed by post hoc test, an increase in serum concentration (1–10%) significantly decreased the uptake of FAM-OL peptide in BBB endothelium and this effect was prominent with progression of incubation time at 12 h (vs. 1 h; Fig. 3b).

### Stability of the internalized active OL peptide

**Fig. 4:**
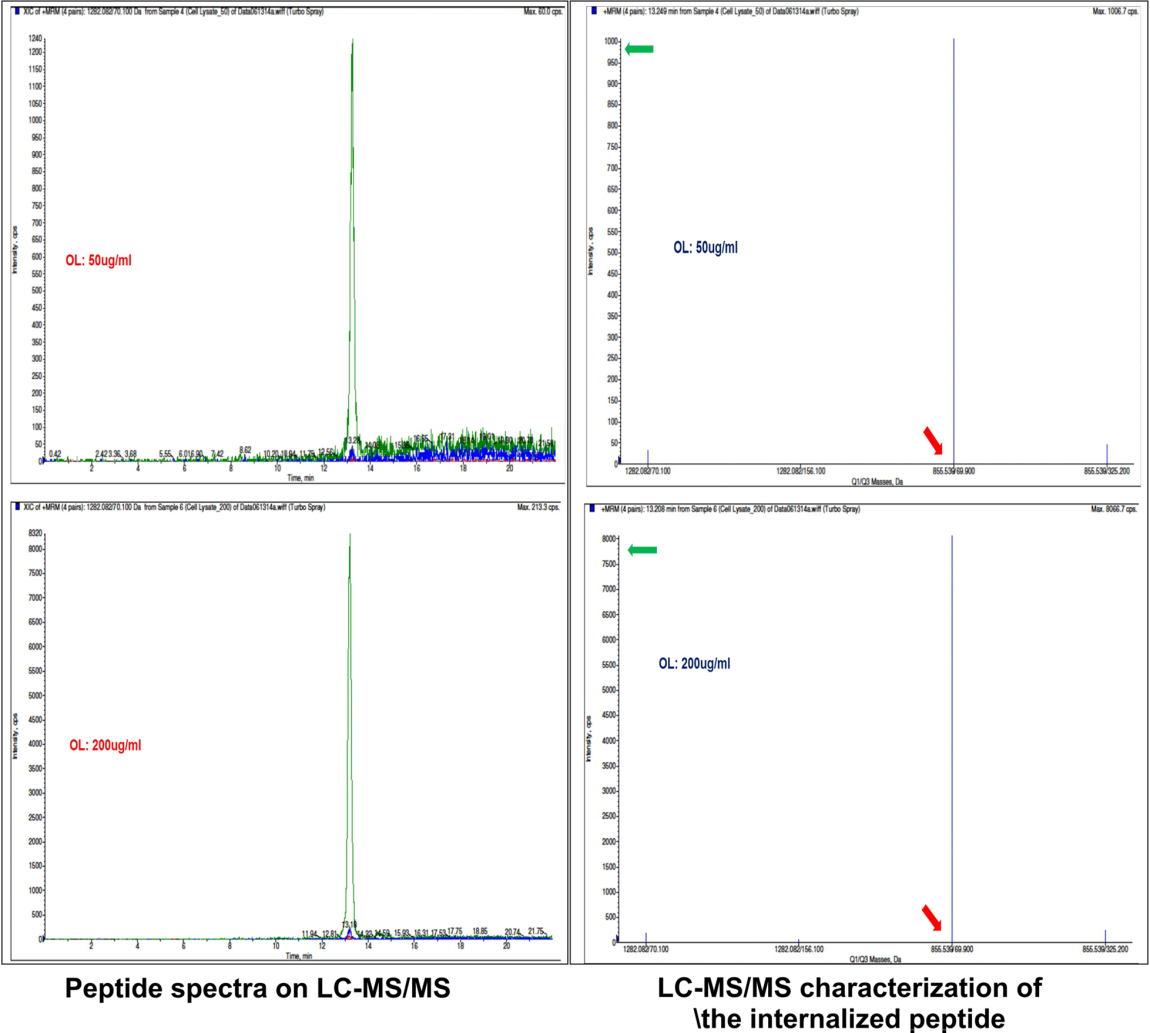
LC–MS/MS (MALDI-TOF) characterization of the internalized peptide in hCMEC/D3 cell line at various concentrations obtained from cell lysates following incubation with the OL (active) peptide for 3 h at 37 °C (N = 4 biological replicates/group pooled from two independent experiments were used for statistical analyses)

Following 3 h incubation of the FAM-OL with hCMEC/D3, the original intact peptide is still detectable. Figure 4 shows detection of the intact FAM-OL tested at two different concentrations (50, and 100 µg/mL) after 3 h incubation with hCMEC/D3 (found m/z = 856, calculated m/z = 861.5). Note that ESI LC–MS has a upper MW limit and this technique for masses above 1200 Da (depending on the instrument to some extent) does not show the molecular ion but m/z demonstrating that FAM-OL did not decompose under the applied experimental conditions.

These data are in a good agreement with previous findings demonstrating increased stability of cyclic peptides toward proteolytic degradation [18].

## Discussion

The CNS is protected and inherently isolated from the systeic circulation by a highly efficient and dynamic interface know as blood–brain barrier. The BBB controls and regulates the passage of substances from the bloodstream into the nervous tissue and maintains the homeostasis of the brain environment [19]. In addition to that, the BBB protects the brain from potentially harmful factors including our own immune system and act as a functional interface through which the brain monitors and exchange signals with the peripheral systems. While the BBB afford selective passage into the CNS of molecules (including nutrients) that are essential for brain function, this creates major obstacles to the passage of drugs in the brain for the treatment of CNS disorders [20, 21].

Different approaches have been developed to improve drug delivery across the BBB. These encompasses the use of nanoparticles, brain permeability enhancers, exosome and other means as described elsewhere {Dong, 2018 #27}. Among the these novel delivery systems, peptide-based drug delivery afford a good degree of selectivity and significant enhancement of drug bioavailability/efficiency at the target site {Wanjale, 2017 #33;Lin, 2019 #32}. Thus, it is not surprising that peptides have received interest as delivery vectors to shuttle drugs across the BBB. These small peptides have several advantages over other delivery systems: they are easy to obtain; can be chemically modified to change their binding affinity and other biological properties (deliver covalently or noncovalently bonded agents including drugs and diagnostic markers); are stable in a broad range of biological conditions; can cross the cellular plasma membranes to deliver their cargo into the cytosol or the nucleus {Ghosh, 2018 #31}. In addition, small peptides are not immunogenic. Among these, cell penetrating peptides are extensively investigated for their inherent potential to deliver oligonucleotide therapeutics across various biological barriers in addition to evading the stability concerns in circulation [4, 22]. Lectins have an interesting biological profile of specifically and selectively binding to vast array of glycans in cell membranes and are widely studied for their drug targeting and delivery at various sites [4, 5, 7]. Recently, OL (the smallest lectin-like peptide), was shown to have low immunogenicity and toxicity potential—known limitations of cell penetrating peptide delivery systems [7, 22].

In this study, we characterized the uptake mechanism and fate of internalized OL peptide (conjugated with FAM) in cellular model of human BBB endothelium, as a part of our long-term goal to develop OL peptide-based systems for brain delivery of siRNA therapeutics. As shown in Figs. 2, 3 and 4, our data revealed a concentration dependent and temperature sensitive cellular uptake of OL peptide in a pattern specific pathway. Given the lack of negative effects on cell viability even at the highest dose tested (200 µg/mL), it is possible to further increase the concentration of the peptide as a drug carrier. Further studies are required to explore the OL peptide and specific glycans at the glycocalyx enriched BBB endothelium [23, 24]. Moreover, our results demonstrate that FAM conjugated OL peptide may evade the lysosomal trapping that would otherwise limit the potential of peptide delivery systems [7].

Further, as shown in Fig. 3b, our results demonstrate a decrease in cellular uptake of FAM-OL peptide with increasing serum concentration in the culture media. These data suggest the serum protein binding potential of FAM conjugated OL peptide, which may implicate that OL peptide could have a longer half-life in systemic circulation. Alternatively, this would also indicate that OL peptide may have reduced internalization due to protein binding. However, the pharmacokinetic profile of OL peptide needs to be assessed in in vivo studies to further confirm our preliminary in vitro. Importantly, we determined the stability of internalized peptide using LC–MS/MS (Fig. 4) and further confirmed an increase in internalized peptide when incubated with increasing concentrations of OL peptide. It should be noted that conjugation of fluorescence probe via PEG-linker to OL also protects the linear N-terminal tail region from proteolytic degradation, further increasing OL stability.

## Conclusions

Our results demonstrate a dose- and sequence-dependent vesicular uptake of FAM conjugated OL peptide by the endothelial cells that is influenced by the protein content of the culture media. As also shown in Fig. 4, it is quite evident that the internalized OL is stable at all concentration tested further supporting the notion that this peptide is likely internalized into the cellular endosome but not the lysosomes. Our data further implicates the potential of OL peptide as a therapeutic carrier for targeted delivery of large molecule drugs as suggested by previous studies; however, additional experimentation in vivo will be necessary to fully assess the viability of OL as a targeted delivery system across the BBB for CNS therapeutics. Concerning the sugar moieties that are targets for the lectin-like peptide it is worth noting that there is a vast array of glycans and as suggested by Rodriguez MC et al., cyclic lectin mimicking peptides bind to glycan complexes on the cell membrane. So, it is possible that OL peptide may bind to the glycocalyx that is abundant on BBB endothelial cell membrane and this need to be further investigated. However, OL is too small to bind the whole glycan, instead it binds terminal monosaccharides. We have previously shown that OL strongly binds to L-fucose and to a lesser degree, to galactose and *N*-acetyl-d-galactosamine [6].

## Methods

### Synthesis of FAM-OL Peptide

Linear peptidyl-resin precursors for naturally occurring cyclic peptide OL, fluorescently labeled analogue, FAM-OL and a control peptide composed of a scrambled OL sequence were synthesized by fluorenylmethyloxycarbonyl (Fmoc)–solid phase peptide synthesis (SPPS) on TentaGel XV RAM resin using an automated peptide synthesizer, as previously described [6]. In brief, amino acid couplings were completed by using fourfold excess of amino acids and coupling reagents 2-(1H-Benzotriazole-1-yl)-1,1,3,3-tetramethyluronium hexafluorophosphate (HBTU)/1-Hydroxybenzotriazole hydrate (HOBt) in the presence of 0.4 M *N*-methylmorpholine (NMM) in *N,N*-Dimethylformamide (DMF). The solid-phase cyclization was achieved by disulfide bridge formation using I_2_ oxidation methodology previously described by us and others [10]. Fmoc-deprotection cycles were carried out using 20% piperidine in DMF solution. After the N-terminal Fmoc-protecting group removal a 20-atom linker, Fmoc-NH-(PEG)_2_-COOH, was coupled using standard HBTU/HOBt coupling methodology, In the next step, the Fmoc-group was removed, and FAM was coupled overnight using 1,3-Diisopropylcarbodiimide (DIC)/HOBt (10 eq of all coupling reagents) in DMF. The final deprotection and peptide cleavage from the resins were carried out with a cleavage cocktail of Trifluoroacetic acid (TFA)/thioanisole/H_2_O (95:2.5:2.5, v/v/v). Analytical RP-HPLC analyses and peptide purifications were performed on 1260 Infinity (Agilent Technologies, Santa Clara, CA) liquid chromatography systems equipped with a UV/Vis detector. For analytical RP-HPLC analysis, a C18 monomeric column (Grace Vydac, 250 × 4.6 mm, 5 mm, 120 Å), 1 mL/min flow rate, and elution method with a linear gradient of 2 → 100% B over 45 min, where A is 0.1% TFA in H2O, and B is 0.08% TFA in CH_3_CN. For peptide purification, a preparative C18 monomeric column (Grace Vydac, 250 × 22 mm, 10 mm, 120 Å) was used. Mass spectrometry was performed on MALDI–TOF Voyager-DE™ STR (Applied Biosystems, Foster City, CA) in reflector-mode using α-cyano-4-hydroxycinnamic acid as a matrix and in positive mode. In all cases, peptide purity was ≥ 95% as determined by RP-HPLC [6, 14]. Figure 1 shows the chemical structures for FAM-conjugated OL (Fig. 1a1) and scrambled peptide sequences (Fig. 1a2).

### Cell culture

All experiments were performed on human brain microvascular endothelial cell line (hCMEC/D3; Millipore Sigma, Massachusetts, US, cat# SCC066), an established and well characterized model of human BBB in vitro [11, 15, 16]. Cells (2.5 × 10^3^/cm^2^; passages 29–32) were cultured on sterile flasks, 96-well plates (for cell viability) and chamber slides (for immunofluorescence imaging) previously coated with Matrigel in HEPES buffered basal endothelial medium (EBM2) supplemented with various growth factors and 5% FBS using Lonza EGM2 MV BulletKit™ [15]. Following 80–90% confluence, cells were subjected to experimental conditions as listed below with serum concentration reduced to 1% FBS. Culture medium’s composition was adjusted (EBM2 medium buffered with HEPES and 1% BSA with no growth factors) to determine the effects of pH and serum (%; protein binding) on the cellular uptake of FAM-OL peptide. Basic pH was maintained with addition of 1 N sodium hydroxide to the medium, while acidic pH was established by addition of phosphoric acid. Cells were lysed with RIPA buffer at the end of treatment with OL peptide and the florescence intensity was measured using BioTek Synergy2 Multimode microplate reader using cellular lysates from cells treated with 0.1% DMSO as controls at 490/520 nm [15].

### Cell viability

Effects of FAM-OL peptide (0–200 µg/mL) on BBB endothelial cell viability were tested by MTT assay. Briefly, following incubation with OL peptide for 3 h in culture media with 1% FBS, cells were rinsed with 200 µL of DPBS for two times and stained with 20 µL of 5 mg/mL MTT for 3 h, as described previously [17]. Following the addition of DMSO, the absorbance at 570 nm was measured using plate reader.

### FAM-OL peptide cellular internalization

HCMEC/D3 cells cultured in chamber slides or 96 well plates were exposed to increasing concentrations of FAM-OL peptide (10–100 µg/mL) solubilized in the culture media (1% FBS) containing 0.1% DMSO (vehicle), at two different temperatures (37 °C and 4 °C). Cells in the negative control group were exposed to scrambled sequence of the peptide (100 µg/mL). Following 3 h, the cells were rinsed with ice-cold HBSS (100–500 µL each well/chamber and two times each) and subsequently prepared for cellular imaging by immunofluorescence (fixed with 4% formalin) or lysed with 0.5% triton X-100 buffer. Internalized peptide was normalized to protein content in the lysates determined by Bicinchoninic acid (BCA) protein assays (as described in the manufacturer’s protocol; Pierce Protein Assay Kit, Thermo Scientific™). To further study the course and fate of the internalized peptide, cells (cultured in EBM2 medium with 1% FBS and no growth factors) were added 10 µM of pHRhodo Red (marker for early to late endosomes) or LysoTracker^®^ Red (marker for lysosome) (Invitrogen) following the addition of FAM-OL peptide and co-imaged after 3 h from addition of trackers using EVOS™ FL Imaging System (digital inverted fluorescence microscope; ThermoFisher—Cat# AMF4300) at 40× magnitude (FAM: 470/22 and 510/42; RFP: 531/40 and 593/40 and DAPI: 357/44 and 447/60; emission and excitation wavelengths respectively).

### LC/MS/MS analysis

After incubation of hCMEC/D3 cells cultured in glass bottom culture dishes with FAM-OL (50 and 200 μg/mL) for 3 h at 37 °C and 1% FBS, cells were centrifuged for 5 min at 4000*g* in a microcentrifuge, following by the pellet washing and resuspension in water [6]. Sonication (20 kHz, 2 × 10 s, Branson Digital Sonifier 250) was used to disrupt cellular membranes and release the cells contents. Lysed samples were centrifuged at 10,000×*g* for 20 min, and supernatant was analyzed by RP-HPLC as described above (1260 Infinity, Agilent Technologies, Santa Clara, CA liquid chromatography systems equipped with a fluorescent detector). Fractions containing FAM-OL were analyzed by LC–MS in reverse phase multiple reaction monitoring (MRM) mode on a Shimazdu LC-20A Prominence HPLC system connected to Absciex 3200 Q Trap MS/MS using a Phenomenex Luna column at 214 nm (5µ C18, 100 Å, 50 × 4.60 mm, linear gradient of 0 → 100% B over 14 min at 0.5 mL/min flow rate, where A is 10 mM ammonium formate and B is 0.1% formic acid in CH_3_CN). MS instrument parameters were spray voltage 5.5 kV, curtain gas 25 psi, source temperature 700 °C, ion source gas 1 70 psi, and gas 2 60 psi. The ion transitions monitored were 578.2/70.2, 578.2/217.2, 600.2/572.2 and 600.2/425.3 with 150 ms dwell time and 5 ms pause time between the transitions. Blank solvent injections were run between each sample to minimize analyte carry-over from one LC–MS/MS run to the next.

### Statistical analyses

Data were reported as mean ± SEM and analyzed for statistical significance using one-way ANOVA, followed by Tukey’s post hoc test, with *p* value set at ≤ 0.05 for significance. For each endpoint, data were collected from two independent experiments with n = 3–5 replicates.

## Data Availability

Supporting data will be made available upon request to the corresponding authors.

## Abbreviations

BBB: blood–brain barrier
OL: odorranalectin
FAM: 5(6)-carboxyfluorescein
CNS: central nervous system
HBTU: 2-(1H-benzotriazole-1-yl)-1,1,3,3-tetramethyluronium hexafluorophosphate
1-HOBt: hydroxybenzotriazole hydrate
NMM: *N*-methylmorpholine
DMF: *N*,*N*-dimethylformamide
SPPS: solid phase peptide synthesis
Fmoc: fluorenylmethyloxycarbonyl protecting group
DIC: 1,3-diisopropylcarbodiimide
TFA: trifluoroacetic acid
MRM: multiple reaction monitoring
